# Microfluidic engineering of immune-competent organs-on-chips and their applications

**DOI:** 10.3389/fimmu.2026.1743806

**Published:** 2026-03-09

**Authors:** Xianwei Liang, Xiangyang Bu, Shuai Yuan, Chengjun Wu, Dongdong Liu, Jiu Deng

**Affiliations:** 1 School of Health and Life Sciences, University of Health and Rehabilitation Sciences, Qingdao, China; 2 Department of Hepatobiliary Surgery, Qingdao Hospital, University of Health and Rehabilitation Sciences (Qingdao Municipal Hospital), Qingdao, China; 3 State Key Laboratory of Biopharmaceutical Preparation and Delivery, Institute of Process Engineering, Chinese Academy of Sciences, Beijing, China

**Keywords:** drug screening, immune microenvironment, immune-competent organ-on-a-chip, immunotherapy, microfluidics

## Abstract

The emerging field of biomimetic microfluidics is advancing the engineering of immune-competent Organs-on-Chips. These systems overcome the constraints of conventional models by using precise microengineering to regulate cellular composition, three-dimensional extracellular matrix architecture, and dynamic biophysical signals. This review summarizes the core design principles, integrating microfluidics, tissue engineering, and biomaterials, that facilitate the reconstitution of physiological and immune-relevant pathological niches. We examine how such tunable control is utilized to model specific immune contexts, including tumor microenvironments for immunotherapy screening, inflammatory processes in barrier organs, and autoimmune disorders. The integration of these chips with patient-derived organoids and multi-omics technologies is emphasized, illustrating how this combined approach provides new mechanistic insights into human immunology. Finally, we consider the translational prospects of these platforms in promoting personalized immuno-medicine and accelerating immunotherapeutic development, while also addressing ongoing challenges in standardization and the incorporation of greater biological complexity.

## 1 Introduction

The physiological function of human organs is closely linked to their dynamic interactions with immune populations (both resident and infiltrating cells) (1–3). This organ-specific immune microenvironment, composed of tissue immune cells, stromal components, and biochemical signaling networks, plays a fundamental role in tissue homeostasis, repair, and disease pathogenesis (4). Understanding the delicate balance within this niche is not only academically relevant but also crucial for deciphering the mechanisms underlying a broad spectrum of conditions, from cancer and autoimmune diseases to infectious processes and immunotherapy responses (5–9). Indeed, the immune contexture of a tissue can influence tumor progression, modulate inflammatory severity, and affect the success of tissue regeneration.

A longstanding challenge, however, lies in the disparity between the complexity of human organ immunology and the capabilities of existing *in vitro* models. Although substantial progress has been made in recapitulating organ biology, most models remain immunologically silent. Conventional two-dimensional (2D) cultures and even advanced three-dimensional (3D) co-culture systems have primarily emphasized parenchymal epithelia, frequently overlooking the regulatory roles of immune-stromal components. While valuable in specific contexts, 3D co-culture systems generally lack a functional vasculature and a complete repertoire of tissue-resident immune cells. Consequently, they fail to emulate key physiological processes such as immune cell recruitment, extravasation, and bidirectional signaling that define organ-level immunity in health and disease. Although animal models offer systemic complexity, they are often hampered by species-specific differences in immune receptor expression, cytokine profiles, and immune system architecture, limiting their clinical translatability and contributing to late-stage drug development failures (10). This widespread omission of faithful immune components constrains the physiological relevance and translational utility of current models, underscoring the need for more predictive preclinical platforms.

The emergence of biomimetic microfluidic platforms, commonly referred to as organ-on-a-chip (OoC) technology, promises to address this gap (11–17). Recently, the improved physiological fidelity of OoCs has gained formal recognition from the United States Food and Drug Administration (FDA), which has endorsed the use of certain alternatives to animal testing, including OoC models, in drug safety and efficacy evaluations (18). These systems are uniquely equipped to overcome previous limitations through core engineering principles such as spatial compartmentalization, continuous perfusion, and the application of physiologically relevant mechanical cues (19, 20). These features provide a robust foundation for building integrated organ-immune units (21–23). For example, microfluidic channels enable the formation of perfusable vascular lumens, permitting direct modeling of immune cell trafficking and extravasation (24). Simultaneously, precisely patterned extracellular matrices support the co-culture of organ-specific parenchymal cells with immune populations in a physiologically relevant 3D architecture (25). Moreover, the incorporation of organ-specific mechanical forces, such as cyclic strain in lung alveolus chips (26), delivers biomechanical inputs known to directly modulate immune cell function. By integrating these elements into a controlled, human-cell-based system, biomimetic chips offer a powerful opportunity to dissect the precise mechanisms through which immune microenvironments regulate organ function and dysfunction.

While recent reviews have broadly chronicled the technological evolution of OoCs and their potential in drug development (11, 27), and methodological studies have detailed specific immune cell integration protocols (23, 28), this review distinguishes itself by focusing on the core design paradigm of “engineered immune microenvironments”. We move beyond discussing the mere assembly of technical components or the inclusion of isolated immune modules. Instead, we emphasize how targeted microfluidic engineering, through the regulation of fluid shear stress, matrix stiffness, and spatial patterning, can be leveraged to actively control immune phenotypes (e.g., polarization, exhaustion, migration). This shift from passive observation to active manipulation enables these platforms to serve as dynamic tools for dissecting complex immune pathologies. Furthermore, we highlight how the convergence of this engineered approach with patient-derived organoids and multi-omics technologies bridges a critical gap: from observing immune phenomena to deciphering their underlying biological mechanisms.

To this end, the review first details the fundamental engineering principles that enable the reconstruction of human immune niches. It then highlights key applications, from modeling tumor-immune landscapes for immunotherapy screening to investigating inflammatory and autoimmune pathologies. The convergence of chip technology with patient-derived organoids and multi-omics for deeper mechanistic insight is examined, followed by a discussion of current challenges and future directions. Collectively, we offer a perspective on how these engineered systems are advancing personalized immunomedicine and refining our understanding of human immunology.

## 2 Fundamental engineering principles and key technologies for immune-competent organ-on-a-chip

**Figure 1:**
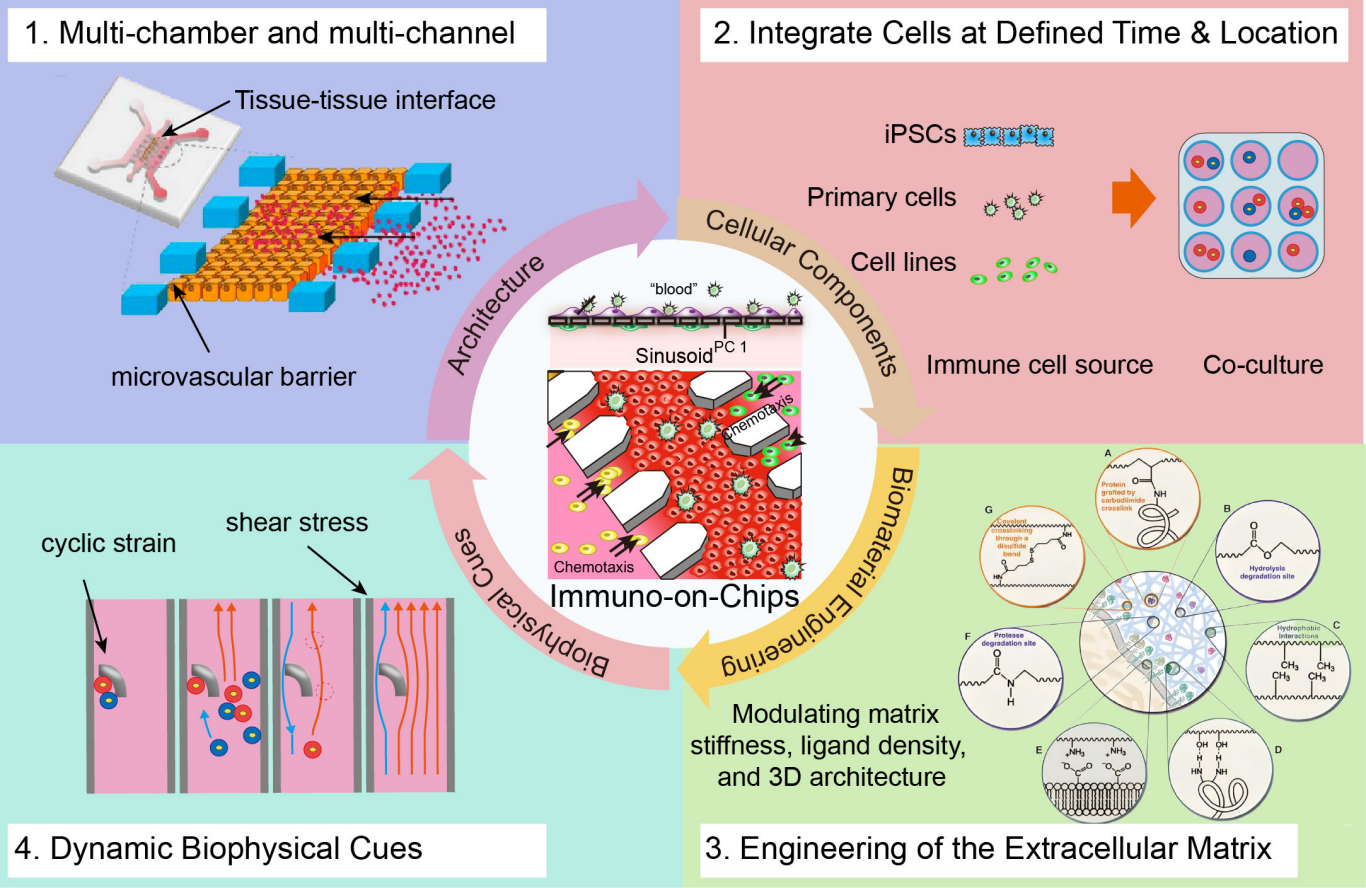
Core engineering strategies for constructing immune-competent organs-on-a-chip. Key pillars include (1): Spatial patterning of biological structures and control over cellular-scale microenvironments through microfluidic architecture [reproduced with permission from an open-access article (24)]; (2) Spatiotemporal engineering of cellular components to mediate immune-specific cell-cell interactions; (3) Programming immune cell behavior via engineered extracellular matrix (ECM) that serves as an active signaling scaffold; (4) Modulation of cell-cell interactions by applying dynamic biophysical cues, such as interstitial flow [reproduced with permission from an open-access article (29)].

Faithfully recapitulating human immunology *in vitro* requires more than simply co-culturing immune cells with parenchymal tissues; it necessitates a systematic engineering strategy to reconstruct the complex physical and chemical framework governing immune cell recruitment, activation, and function. The integration of microfluidic engineering, cell biology, biomaterials science, and biomechanics has enabled the development of sophisticated Immune-Competent Organ-on-a-Chip (IcOC) platforms (**Figure 1**) (29). This section outlines the core design principles of these bioengineered microphysiological systems, focusing on the strategic combination of architectural design, cellular components, extracellular matrix (ECM), and dynamic biophysical cues.

### 2.1 Design of microfluidic architecture

A key advantage of OoC technology is its capacity to spatially pattern biological structures and control fluidic microenvironments at cellular scales. This physical control is essential for mimicking anatomical sites where immune responses take place, such as vessel-tissue interfaces. Two principal technologies for microfluidic architecture design, modeling tissue-tissue interfaces and engineering microvascular barriers with controlled perfusion, are outlined below.

#### 2.1.1 Multi-chamber and multi-channel systems to model tissue-tissue interfaces

*In vivo*, immune responses are often orchestrated across specific anatomical interfaces, for example, between vascular endothelium and organ parenchyma, or intestinal epithelium and underlying mucosal immune cells. Microfluidic chips replicate these interfaces using parallel or vertically stacked microchannels and chambers. For instance, Rennert et al. incorporated monocyte-derived macrophages into the endothelial layer of a hepatic sinusoid model to simulate the immune functions of resident Kupffer cells in the liver (30). Similarly, Yuan et al. designed immune cell chambers on the vascular side of an endothelial layer, enabling immune cell migration into a liver chip via chemotaxis during inflammation (13).

A common design is the three-channel architecture, featuring a central “tissue” chamber, filled with an ECM hydrogel such as collagen I or fibrin and seeded with organ-specific parenchymal cells (*e.g.*, hepatocytes, alveolar epithelial cells) and stromal cells (*e.g.*, fibroblasts), flanked by two “vascular” channels lined with human endothelial cells to form a functional lumen. For example, Deng et al. developed a three-channel microphysiological system to study immune-liver interactions, introducing immune cells into lateral channels to model inflammatory infiltration and assess immune-mediated hepatotoxicity of troglitazone and *Polygonum multiflorum Thunb* (24, 31).

This configuration establishes a well-defined, quantifiable interface. Under inflammatory stimuli (*e.g.*, TNF-α, IL-1β), immune cells perfused through the vascular channel can be observed in real time undergoing extravasation steps: rolling, firm adhesion, diapedesis, and migration into the tissue compartment. Spatial segregation also allows independent control of each chamber’s microenvironment, for instance, modeling a localized inflammatory focus in the tissue while maintaining vascular homeostasis. This capability is crucial for dissecting bidirectional signaling between tissue and immune cells (32).

#### 2.1.2 Engineering of microvascular barriers and controlled perfusion

**Figure 2:**
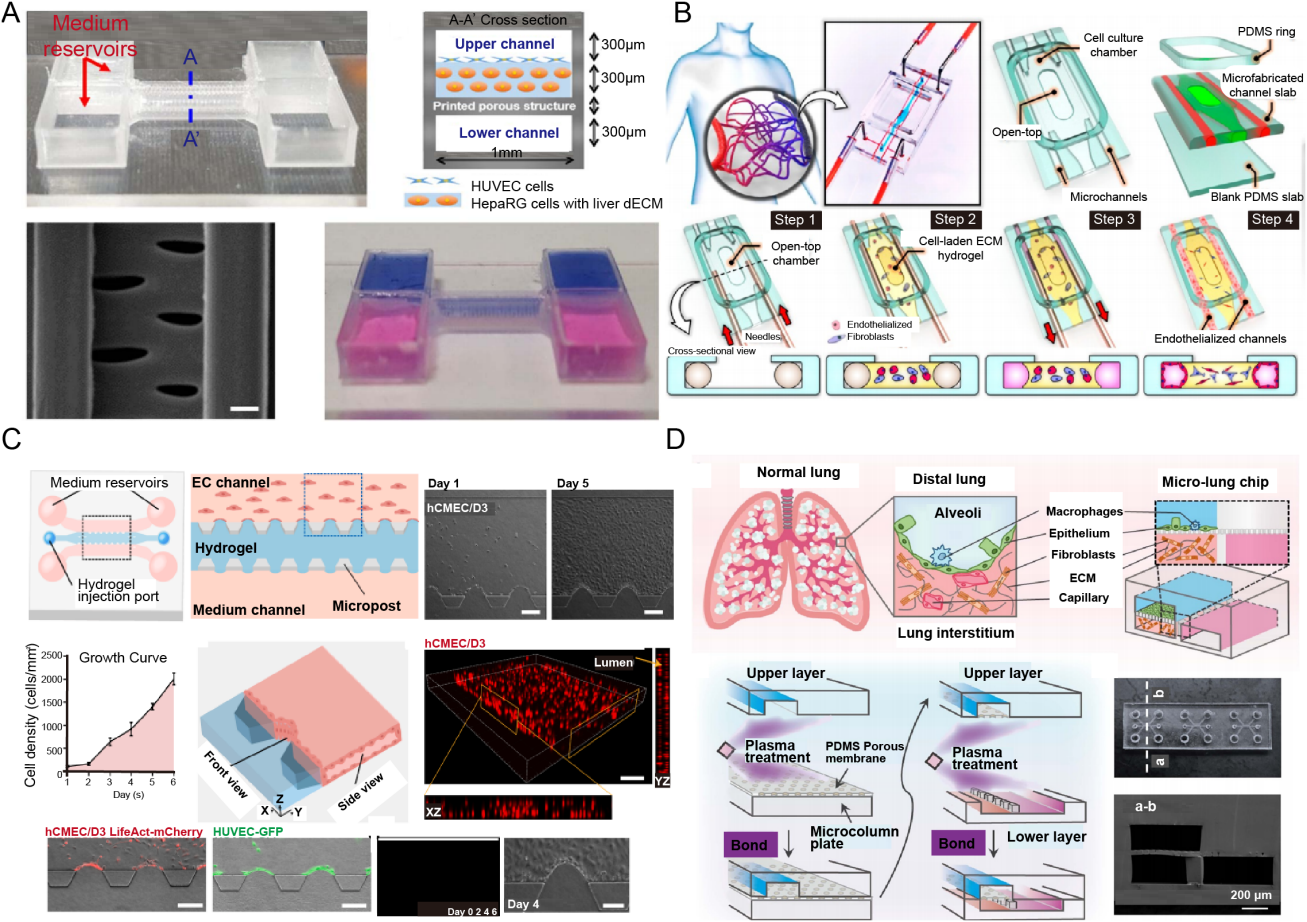
Engineering strategies for reconstructing complex immune-competent organs-on-a-chip. **(A)** 3D−printed microvascular barriers with controlled perfusion to model immune cell trafficking and vascular permeability under inflammatory conditions [reproduced with permission from an open-access article (35)]; **(B)** Self−assembled capillary networks under defined biochemical cues to study angiogenic−immune crosstalk and endothelial−immune cell interactions [reproduced with permission from an open-access article (36)]; **(C)** Spatiotemporal co−culture of parenchymal, stromal, and immune cells to recapitulate neuroinflammation−mediated BBB breakdown and evaluate immunomodulatory drug efficacy [reproduced with permission from an open-access article (37)]; **(D)** Integration of dynamic biophysical cues such as interstitial flow to investigate mechano−immune regulation of cell migration and cytokine dispersion [reproduced with permission from an open-access article (38)].

A functional, perfusable microvasculature is fundamental for studying systemic immunity. Current engineering strategies fall mainly into “top-down” and “bottom-up” approaches. Top-down methods use soft lithography or 3D printing to create predefined channel structures that are subsequently seeded with endothelial cells to form lumenized vessels. This approach ensures high reproducibility, geometric control, and ease of integration. For instance, Boussommier-Calleja et al. engineered a 3D vascularized microfluidic model with three distinct hydrogel channels to study monocyte transmigration across human microvessels (33, 34). Lee et al. designed a 3D liver-on-a-chip with a dual-flow system using 3D-printed vascular and biliary channels; HUVECs cultured on decellularized ECM bioink formed adherens junctions and liver sinusoid-like structures (**Figure 2A**) (35). Controlled perfusion in such models enables application of physiological shear stress on the endothelium, a key mechanical cue regulating inflammatory activation and immune cell adhesion.

Bottom-up strategies involve seeding endothelial cells, often with pericytes or fibroblasts, into a matrix-filled chamber, where they spontaneously self-assemble into 3D capillary networks under appropriate biochemical cues. These self-assembled capillaries better mimic the morphology, size, and barrier function of native capillaries, often supporting more physiological permeability and immune cell transendothelial migration (37, 38). For example, Paek et al. established a method for culturing perfusable 3D microvascular networks *in vitro* that closely recapitulate vasculogenesis (**Figure 2B**) (36). Although integrating controlled perfusion with self-assembled networks remains technically challenging, it is vital for modeling dynamic immune surveillance and inflammatory recruitment with high physiological relevance. Lee et al., for instance, developed a self-organized tumor microenvironment (TME) array with a bioprinted HUVEC barrier surrounding breast cancer spheroids, formed via a liquid-hydrogel interface with capillary pinning (39).

### 2.2 Engineering cellular components

While the microfluidic chip provides a finely tuned physical environment, it is the engineered cellular components that drive immune responses. This requires spatiotemporal precision: the strategic integration of specific cell types at defined times and locations to choreograph the cell-cell interactions underlying immunity.

#### 2.2.1 Co-culture strategies: spatiotemporal introduction of parenchymal, stromal, and immune cells

Mere mixing of different cell types is inadequate to emulate the complex cellular choreography of an immune response. Successful co-culture depends on the temporal sequence and spatial localization of cell seeding. A common approach is to first establish a stable endothelial barrier and parenchymal tissue architecture before introducing immune cells, mimicking physiological recruitment from circulation. For instance, by introducing neutrophils and T cells into a biomimetic hepatic sinusoid chip, Yuan et al. developed an immunocompetent liver chip that modeled immune cell migration and stress-responsive behaviors induced by chemokines (13).

The immune compartment’s diversity must also be considered. Beyond basic T cell/macrophage co-cultures, incorporating tissue-resident macrophages, dendritic cells, neutrophils, innate lymphoid cells, and B cells is essential to recapitulate a full-spectrum immune response (40). For example, microglia, the resident immune cells of the brain, are often included in brain chips to introduce immune functionality. Zeng et al. developed a blood-brain barrier (BBB) model with endothelial cells, astrocytes, and microglia to study neuroinflammation-induced BBB disruption and assess drug efficacy (**Figure 2C**) (37). Moreover, stromal cells such as fibroblasts are not merely passive structural elements; they actively shape the immune milieu by secreting cytokines and chemokines and remodeling the ECM, thereby influencing immune polarization, for example, toward pro-fibrotic or immunosuppressive states. A systematic, sequential co-culture strategy is therefore essential for reconstructing a functional immune niche.

#### 2.2.2 Sources of immune cells: cell lines, primary cells, and stem cell-derived progeny

The choice of immune cell source significantly affects the physiological relevance and reproducibility of IcOC models. Immortalized cell lines (*e.g.*, THP-1 monocytes, Jurkat T cells) are readily accessible and easy to culture but often have altered genotypes and phenotypes, restricting their utility to preliminary screening. For example, Qiang et al. used THP-1-derived macrophages to mimic splenic red pulp macrophages in a spleen-on-a-chip model, revealing enhanced adhesion and phagocytosis of sickle cell disease red blood cells under hypoxia (41). Primary human cells isolated from peripheral blood (PBMCs) or tissues offer high physiological fidelity and are the gold standard for patient-specific disease models. For instance, Lu et al. demonstrated in a vasculature-on-a-chip infected with SARS-CoV-2 that introducing PBMCs exacerbated cytokine-induced endothelial dysfunction, revealing monocyte, endothelium crosstalk in hyperinflammation (42). However, primary cells exhibit donor variability, limited expansion capacity, and finite lifespans. Human induced pluripotent stem cells (iPSCs) are emerging as a transformative alternative. They can be expanded indefinitely and differentiated into diverse immune cell types (e.g., macrophages, microglia, T cells) while retaining the patient’s genetic background. This supports the creation of scalable, personalized, and ethically acceptable “immune-system-on-a-chip” models, particularly valuable for studying genetic immunodeficiencies and personalized cancer immunotherapies. Narasipura et al., for example, established a brain organoid model incorporating iPSC-derived microglia-like cells, which differentiated spontaneously within a neurodevelopmental context when co-cultured with iPSC-derived hematopoietic progenitor cells (HPCs) (43).

Beyond the initial selection of cell sources, their sustained performance during on-chip culture is critical for faithfully recapitulating acute or chronic immune processes. While cell lines offer convenience, their genotypic and phenotypic instability under prolonged perfusion limits their utility in modeling dynamic, long-term functions such as T-cell activation and exhaustion. Primary cells provide high physiological fidelity but often exhibit declining activity after several days in culture, compounded by donor-dependent variability that complicates extended studies. In contrast, iPSC-derived immune cells present a promising avenue for longer-term cultures due to their expandability and genetic relevance, although maintaining stable, functionally mature phenotypes over weeks remains an area of active investigation.

The requisite culture duration is dictated by the biological question—from hours to days for acute inflammatory responses to several weeks for modeling chronic T-cell exhaustion or tumor-immune editing. Advances in chip design, incorporating optimized perfusion, 3D matrices, and physiological mechanical cues, now support immune-parenchymal co-cultures for up to several weeks, enabling the observation of temporal immune evolution. Nonetheless, preserving the consistent functional attributes of specific immune subsets, such as cytotoxic T-cell activity or regulatory T-cell suppression, across different cell sources and extended timelines remains a pivotal technical challenge for the field.

### 2.3 Biomaterial engineering of the extracellular matrix

The ECM is not a passive scaffold but an active signaling component that critically influences cell fate and immune function. Engineering the ECM is therefore essential for instructing immune cell behavior.

#### 2.3.1 Hydrogel selection and application

Natural hydrogels such as collagen I and Matrigel are widely used due to their inherent bioactivity and cell-adhesive motifs, which support cell growth and migration. Calò et al. reported that collagen concentration regulates neutrophil migratory behavior during infection by affecting transmigration efficiency, speed, and travel distance (44). However, natural hydrogels suffer from batch-to-batch variability and limited mechanical tunability. Synthetic hydrogels based on poly(ethylene glycol) (PEG) offer highly defined and customizable physicochemical properties with excellent reproducibility. By incorporating cell-adhesive peptides (*e.g.*, RGD) or matrix metalloproteinase (MMP)-sensitive cleavage sites, “designer ECMs” with tailored biofunctionality can be created. For example, modifying the method of RGD presentation (e.g., using CSP-RGD) allows independent control over cell adhesion without significantly altering physical properties such as stiffness or swelling ratio (45). Hybrid hydrogels, such as methacrylated hyaluronic acid (MeHA) or gelatin, are increasingly popular, combining the bioactivity of natural materials with the tunability of synthetic systems (46, 47).

#### 2.3.2 Modulating matrix stiffness, ligand density, and 3D architecture

The physical and chemical properties of the ECM profoundly influence immune cells. Matrix stiffness, for instance, is a critical parameter. In tumor-on-a-chip models, stiffened ECMs mimicking fibrotic tumors promote macrophage polarization toward a pro-tumoral (M2) phenotype and impair T cell cytotoxicity (48, 49). By modulating hydrogel crosslinking density, the independent effect of stiffness on immune cell differentiation and function can be systematically studied. For example, Wei et al. reported that the density of functional blood vessels generated by implanted collagen hydrogel could be doubled through a combined strategy. This approach mitigated the immune response from cross-linkers while utilizing “spacers” to create an intravascular hypoxic environment (50). Similarly, the density and spatial presentation of adhesive ligands (*e.g.*, ICAM-1, VCAM-1 mimetics) directly govern immune cell migration patterns and speeds (51). Finally, the 3D topology of the ECM, including pore size, fiber alignment, and degradability, provides physical guidance cues that direct immune cell migration and shape intercellular interactions (52).

### 2.4 Integration of dynamic biophysical cues

The immune system functions within a dynamic mechanical environment, and neglecting these forces yields incomplete models (53, 54). Key biophysical cues include: Interstitial flow, the slow movement of fluid through tissue interstitium, not only transports nutrients and oxygen but also shapes immune responses (55, 56). By washing away local chemokines, it establishes long-range chemical gradients that guide dendritic and T cell migration. The resulting fluid shear stress can also activate mechanosensitive pathways, influencing cell alignment, gene expression, and cytokine secretion (57). Cyclic mechanical strain, experienced by organs such as the lungs and intestines during breathing and peristalsis, is physiologically critical (58–60). Studies using lung-alveolus-chips have shown that breathing motions enhance neutrophil and monocyte recruitment during infection or inflammation (61, 62). The absence of these cues leads to attenuated and physiologically irrelevant immune responses. For instance, Xia et al. engineered a micro-lung chip featuring an epithelium-interstitium tissue unit to establish a controlled immune environment containing only macrophages. They discovered that macrophages exacerbated inflammation and fibrosis, as evidenced by comparing bleomycin (BLM)-treated microchips with and without macrophages (**Figure 2D**) (61, 62). Vascular shear stress exerts direct effects on both endothelial and immune cells (63). Physiological laminar shear stress promotes an anti-inflammatory endothelial state, while disturbed flow, common at arterial branches, triggers endothelial activation and leukocyte adhesion (64). Flow also applies physical forces to circulating immune cells, modulating their rolling behavior and activation thresholds.

In summary, the systematic integration of dynamic biophysical cues, interstitial flow, cyclic strain, and vascular shear stress, together with microfluidic architecture, cellular components, and ECM engineering, enables the construction of sophisticated, biomimetic human tissue models on a chip (65, 66). These platforms provide unprecedented opportunities to dissect immune microenvironment mechanisms with high precision and human relevance, supporting more predictive drug development and personalized medicine. Ongoing challenges include improving system complexity and throughput while ensuring robustness and standardization for broader adoption (**Table 1**).

**Table 1:** Mapping between design dimensions and immune responses in immune-competent organ-on-a-chip platforms.

Design dimension	Regulated immune response	Mechanism & design strategy	Key findings & translational implications
Microvascular Geometry (24, 34)	Cell Rolling/Adhesion	Channel dimensions, branching angles, and surface topography dictate shear stress profiles, influencing immune cell margination and activation on endothelium.	Geometrically complex regions promote monocyte adhesion and transendothelial migration, simulating inflammatory hotspots.
	Transendothelial Migration	Multi-channel designs (e.g., tri-channel chips) provide a well-defined endothelium-tissue interface for visualizing sequential migration steps.	Real-time observation of immune cell extravasation (rolling, adhesion, diapedesis) under TNF-α stimulation, applicable for drug-induced hepatotoxicity assessment.
Cellular Source & Composition (43, 67)	Immune Cell Polarization	iPSC-derived immune cells retain patient-specific genetic background, enabling modeling of individualized immune phenotypes.	Microglia exhibit activated phenotypes within neuroinflammatory contexts, useful for modeling neuroimmune disorders.
	T Cell Exhaustion	Co-culture of patient-derived T cells with autologous tumor organoids models T cell dysfunction upon chronic antigen exposure.	Enables study of personalized T cell-adipocyte interactions in metabolic immunology.
ECM Stiffness & Composition (44, 48)	Macrophage Polarization	Stiff collagen matrices mimicking fibrotic tumor microenvironments promote a pro-tumoral (M2) polarization.	Identifies CaMKK2 signaling as a mediator of stiffness-induced M2 polarization, suggesting matrix targeting as an immunotherapeutic strategy.
	Neutrophil Migration	Collagen concentration regulates migration speed and directionality, impacting efficiency of response to infection.	High collagen density impedes neutrophil migration, highlighting the role of matrix composition in early immune responses.
Interstitial Flow & Shear Stress (42, 57)	Dendritic Cell & T Cell Chemotaxis	Interstitial flow establishes long-range chemokine gradients, guiding directional migration of immune cells.	Interstitial flow modulates T cell egress and stromal remodeling, simulating immune homeostasis in lymph nodes.
	Endothelial Immune Activation	Physiological laminar flow suppresses endothelial activation, while disturbed flow promotes inflammatory cytokine expression and leukocyte adhesion.	Aberrant shear stress exacerbates endothelial inflammation, implicating hemodynamics in immune dysregulation.
Mechanical Strain (68, 69)	Neutrophil & Monocyte Recruitment	Mimicking breathing motions in alveolar chips enhances immune cell trafficking across the endothelial barrier.	Cyclic mechanical strain suppresses viral replication and potentiates innate immune responses, underscoring the role of mechanical cues in lung immunity.
	Epithelial Barrier Function & Immune Regulation	Application of peristaltic strain in gut-on-a-chip models enhances epithelial barrier integrity and modulates mucosal immunity.	Mechanical strain improves epithelial barrier and suppresses bacterial overgrowth and inflammation.
Spatial Co-culture Configuration (25, 70)	Immune-Tumor Crosstalk	Barrier-free multi-compartment chips facilitate direct cell-cell contact, simulating *in situ* immune infiltration.	High-concentration collagen matrix enhances tumor invasiveness while paradoxically suppressing immune infiltration, highlighting the importance of matrix control in immunotherapy.
	Modeling Autoimmune Attack	Multi-layered co-culture (e.g., neurons, glia, immune cells) enables modeling of targeted tissue damage in autoimmunity.	Autoantibodies and immune cells synergistically drive synovitis, providing a platform for screening autoimmune therapeutics.

## 3 Engineering specific immune microenvironments and their applications

The distinctive strength of IcOC platforms lies in their adaptability for modeling specific physiological and pathological niches. By applying the core engineering principles described in Section 2, researchers are advancing from generic models to customized microenvironments that replicate the unique immunological characteristics of different organs and diseases. This section examines the transformative applications of these platforms in oncology, barrier organ immunology, and autoimmunity.

### 3.1 The tumor immune microenvironment

**Figure 3:**
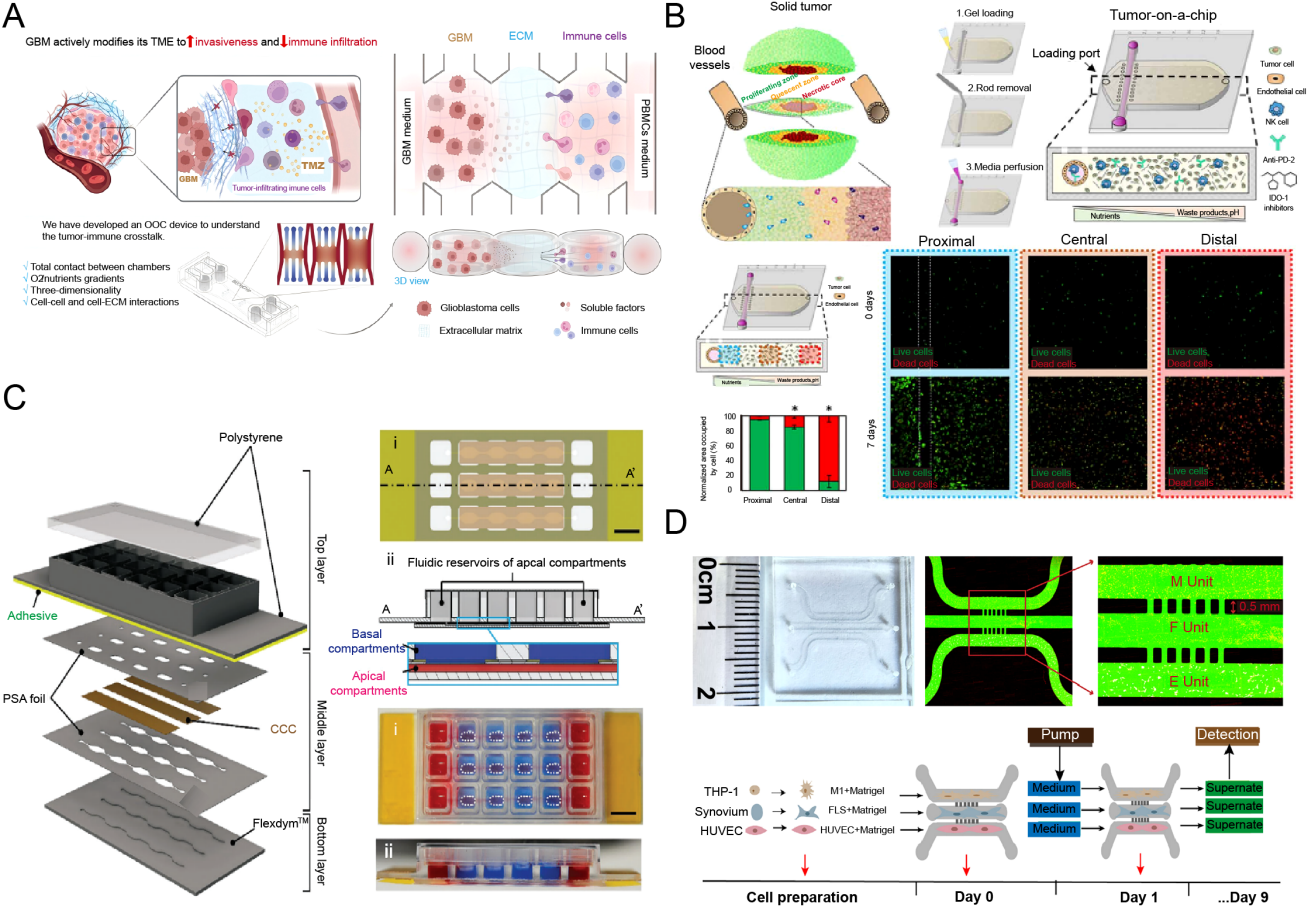
Engineered models of human immune microenvironments for disease modeling and therapeutic screening. **(A)** An engineered tumor immune microenvironment (TIME) to dissect immune checkpoint dynamics and tumor−mediated T−cell suppression for mechanistic insight and drug discovery [reproduced with permission from an open-access article (25)]. **(B)** A perfusable TIME to track real−time immune infiltration, exhaustion markers, and metabolic adaptation within the tumor niche [reproduced with permission from an open-access article (71)]. **(C)** A gut−barrier model to elucidate how host−microbe interactions drive immune−mediated barrier dysfunction in inflammatory bowel disease [reproduced with permission from an open-access article (72)]. **(D)** A microphysiological model of target tissue to deconstruct autoantigen−specific T−cell responses and tissue damage in autoimmune pathogenesis [reproduced with permission from an open-access article (73)].

The TIME represents a complex, dynamic, and predominantly immunosuppressive network of intercellular communications. The tumor immune microenvironment (TME) is a dynamic ecosystem crucial in cancer progression and therapy response. It comprises diverse immune cells, cytokines, and stromal components. Their interactions can either suppress or promote anti-tumor immunity. Understanding the TME is vital for developing immunotherapies, predicting treatment outcomes, and overcoming resistance, making it a central focus in modern oncology. The limited capacity of static *in vitro* co-cultures and animal models to recapitulate human pathophysiology often leads to a failure in predicting immunotherapy outcomes, thereby creating an urgent demand for human-relevant models. Engineering the TIME *in vitro* is thus crucial to elucidate underlying mechanisms and advance therapeutic discovery. Illustrating this potential, Bayona et al. introduced an innovative multi-chamber microfluidic device that eliminates physical barriers, a key limitation of existing platforms (**Figure 3A**) (25). This system facilitates direct tumor-immune interactions and sustains native immune cell infiltration patterns. A pivotal finding from their work is that a high-concentration collagen matrix potentiates tumor invasiveness while paradoxically suppressing immune cell infiltration. Based on these findings, here we will discuss how engineered immune microenvironments are being leveraged to dissect tumor biology and improve immunotherapy screening.

#### 3.1.1 Chip models for immunotherapy screening

OoC platforms are being developed as sophisticated “clinical trials-on-a-chip” for evaluating novel immunotherapies (74, 75). A major advance is the integration of a human vasculature, which is essential for assessing cell-based therapies such as Chimeric Antigen Receptor (CAR)-T cells (76). For example, a vascularized tumor chip can be constructed by seeding a tissue chamber with patient-derived tumor spheroids or organoids in a stromal ECM, adjacent to an endothelial-lined vessel (77, 78). When CAR-T cells are perfused through the vascular channel, researchers can quantitatively monitor key steps of the anti-tumor response in real time: T cell adhesion to the endothelium, extravasation into the tumor, tumor cell killing, and potential cytokine release. This offers unique kinetic and mechanistic insights not attainable through endpoint animal studies.

Similarly, IcOCs enable the study of resistance mechanisms to immune checkpoint inhibitors (*e.g.*, anti-PD-1/PD-L1 antibodies) (79). A model incorporating tumor cells, myeloid-derived suppressor cells (MDSCs), and tumor-infiltrating lymphocytes (TILs) can test whether PD-1 blockade reverses T cell exhaustion in a suppressive microenvironment. The system can also be perturbed with cytokines or small molecules to identify co-targets that enhance the efficacy of checkpoint inhibition.

#### 3.1.2 Investigating intratumoral immune cell infiltration, exhaustion, and metabolic crosstalk

The controlled geometry of OoCs facilitates systematic study of the physical and biochemical barriers to immune cell infiltration. Gradients of oxygen and nutrients can be established across a tumor chamber, enabling investigation of how these conditions alter the secretome of cancer and stromal cells and influence immune cell migration and function (72, 73, 80, 81).

Moreover, the spatiotemporal dynamics of T cell exhaustion can be tracked over time, For example (**Figure 3B**) (71). Repeated exposure of T cells to tumor antigens in the chip models the progressive loss of T cell function, which can be correlated with upregulation of exhaustion markers such as PD-1, TIM-3, and LAG-3. The platform also provides a unique view of metabolic competition within the TIME. High metabolic activity of tumor cells can deplete nutrients (*e.g.*, glucose, tryptophan) and accumulate immunosuppressive metabolites (*e.g.*, lactate, adenosine), directly inhibiting T cell function. IcOCs allow real-time monitoring of metabolite changes and testing of metabolic interventions to restore anti-tumor immunity.

### 3.2 Inflammatory responses in barrier organs

Barrier organs such as the gut and lung serve as primary interfaces with the external environment, and their immune dysregulation underlies numerous diseases.

#### 3.2.1 Gut-on-a-chip: host-microbe interactions and inflammatory bowel disease

The gut is a central immune organ where gut-associated lymphoid tissue interacts directly with the microbiome. This barrier organ constantly encounters external stimuli and hosts a dense microbial community that helps maintain a balance between tissue homeostasis and pathogenic challenge (82). Immune cells such as macrophages, T cells, and dendritic cells reside in the lamina propria, a connective tissue layer underlying the epithelium. Advanced Gut-on-a-Chip models co-culture intestinal epithelial cells (*e.g.*, Caco-2 or primary organoids) with vascular endothelium, immune cells (*e.g.*, PBMCs or dendritic cells), and a living microbiome (83–85). Applying cyclic mechanical strain to mimic peristalsis is essential, as it improves epithelial barrier function and mucus production, creating a more physiologically relevant interface (68, 86). These models have been used to study the breakdown of intestinal barrier integrity and the onset of inflammation in IBD (**Figure 3C**) (72). Introduction of patient-derived CD4^+^ T cells from Crohn’s disease patients enables modeling of T cell-mediated epithelial injury, while specific pathobionts can trigger neutrophilic inflammation. This platform allows dissection of how bacterial strains modulate host immunity and supports testing of anti-inflammatory therapies in a human context.

#### 3.2.2 Lung-on-a-chip: pathogen infection, allergic asthma, and fibrosis

The Lung-on-a-Chip model, which includes an alveolar epithelium exposed to air, a microvascular endothelium, and cyclic strain to mimic breathing, has provided key insights into lung immunity (26, 28, 62). These systems typically incorporate an air–liquid interface (ALI) in a two-channel device, where lung alveolar epithelial cells and pulmonary microvascular endothelial cells are cultured on opposite sides of a porous membrane to simulate the epithelial barrier (87, 88). For instance, Bai et al. used a human alveolus-chip with cyclic breathing motions to show that physiological stretching suppresses viral replication by activating innate immune responses in epithelial and endothelial cells (69). The platform has also been used to model influenza and bacterial infections, demonstrating that breathing motions enhance pathogen uptake and promote neutrophil and monocyte recruitment across the endothelium (28, 89, 90).

In allergic asthma studies, such chips enable analysis of sensitized airways, featuring thickened, mucus-producing epithelium and embedded mast cells, in response to allergens, allowing measurement of histamine and inflammatory cytokine release. Sun et al. designed a bronchiole-on-a-chip with a compact stretching system that mimics various breathing patterns and observed elevated secretion of IL-6 and IL-8 under asthmatic conditions (91). Similarly, Benam et al. developed a “small airway chip” in which IL-13 stimulation induced goblet cell hyperplasia, mucus overproduction, and impaired ciliary function, recapitulating key asthma features (26).

Furthermore, the Lung-on-a-Chip serves as a powerful tool for studying pulmonary fibrosis and drug screening. By introducing pro-fibrotic stimuli such as TGF-β and incorporating immune cells, the system can model the interplay between immune activation, involving macrophages and T cells, and fibroblast proliferation that drives fibrosis (92–94). This human-relevant platform supports screening of anti-fibrotic compounds and elucidation of underlying molecular mechanisms. In summary, the Lung-on-a-Chip not only recapitulates core lung pathophysiology and immune responses but also significantly advances the exploration of disease mechanisms and identification of novel therapies.

#### 3.2.3 Blood-brain barrier chip: neuroinflammation and autoimmune disease

The blood-brain barrier is a selective interface formed by brain microvascular endothelial cells, astrocytes, and pericytes, which tightly regulates immune cell entry into the central nervous system (CNS) (95). BBB-chips that recapitulate this multicellular structure are used to study neuroinflammation (37, 96). In multiple sclerosis models, researchers can perfuse myelin-reactive T cells through the vascular channel and observe their activation by resident antigen-presenting cells and subsequent breach of the BBB, a key step in disease pathogenesis (97). These chips can also model how systemic inflammation, mediated by circulating inflammatory cytokines, affects BBB integrity and CNS function.

### 3.3 Pathogenesis of autoimmune diseases

Organ-on-a-Chip platforms enable denovo construction of target tissues to study the initial phases of autoimmune pathogenesis, allowing precise modeling of disease-specific mechanisms. For example, Palma et al. described a joint-on-a-chip platform that recapitulated a patient-derived rheumatoid arthritis (RA) model and evaluated interactions among different immune cell subsets involved in RA (70). In the context of RA, a Synovium-on-a-Chip can be engineered by co-culturing fibroblast-like synoviocytes (FLS) with a microvascular endothelium. Introducing autoantibodies from RA patients (e.g., anti-citrullinated protein antibodies) along with monocytes and T cells triggers pathogenic events such as immune complex formation, complement activation, endothelial activation, and immune cell infiltration into the synovium (**Figure 3D**) (73, 98). Subsequently, activated FLS display an aggressive phenotype, proliferating abnormally and secreting matrix-degrading enzymes and pro-inflammatory cytokines. This platform provides an ideal setting for monitoring these processes quantitatively and testing biologics against specific cytokines or signaling pathways.

Beyond modeling barrier function, more complex systems can recreate the actual targets of autoimmune attacks (99). For multiple sclerosis, this involves modeling the assault on the myelin sheath. By co-culturing oligodendrocytes with neurons and astrocytes in a 3D matrix, researchers can establish a microenvironment where myelinated axons develop. Introducing myelin-reactive T cells, macrophages, or microglia into this system enables direct observation and quantification of demyelination and axonal damage (100). This setup is valuable for investigating the contributions of different immune subsets to neural injury and for screening neuroprotective and remyelination promoting therapies. The engineering of specific immune microenvironments on a chip offers a powerful, reductionist approach to deconstruct human immunology. By providing controlled, human-relevant, and mechanically active models, these innovative platforms effectively bridge the gap between traditional *in vitro* assays and *in vivo* studies, accelerating both fundamental discovery of disease mechanisms and the development of new therapeutic strategies.

## 4 Frontier convergence and technological innovation

The full potential of IcOC platforms is achieved not in isolation, but through their strategic integration with other advanced biological and analytical technologies. This synergy establishes a powerful feedback loop: OoCs provide a physiologically relevant, human-based context for experimental observations, while emerging tools such as patient-derived organoids and high-resolution multi-omics deliver deep mechanistic insights. This section examines how these integrations are transforming IcOCs from advanced cellular models into predictive, personalized, and highly quantitative platforms for immunological research.

### 4.1 Integration with patient-derived organoids

Organoids, self-organizing three-dimensional structures derived from pluripotent or adult stem cells, effectively mimic the complex functions of human organs, making them valuable platforms for studying organ development, homeostasis, and disease mechanisms (101, 102). These 3D models reproduce key aspects of cellular heterogeneity and tissue architecture, yet they often lack integrated immune components and functional vasculature, limiting their physiological relevance. In contrast, organ-on-a-chip systems excel at providing precisely these elements, including perfusable vascular channels and immune cell interactions. Thus, combining OoC technology with patient-derived organoids represents a paradigm shift toward creating personalized human immunology models *in vitro*. For example, Rogal et al. developed a microphysiological *in vitro* model of human white adipose tissue (67). Their customized, vascularized microfluidic platform facilitates the self-assembly of a complex three-dimensional tissue comprising mature adipocytes, endothelial barriers, adipose tissue macrophages, and other key cellular components. This model recapitulates essential adipose tissue functions, including energy storage, metabolism, and endocrine and immune regulation. Moreover, this precise, bottom-up engineering approach enables the generation of numerous reproducible samples from a single donor, thereby minimizing inter-donor variability and paving the way for personalized medicine.

A particularly promising application of this integrated approach is the construction of autologous tissue-immune models. In oncology, for example, a patient’s tumor organoid can be embedded in the tissue compartment of a chip while their own immune cells, such as peripheral blood mononuclear cells or engineered CAR-T cells, are introduced into the adjacent vascular channel. This “patient-on-a-chip” strategy preserves the individual’s unique genetic and phenotypic disease profile within a controlled yet dynamic microenvironment. It enables direct observation of patient-specific T cell-tumor interactions, including T cell infiltration and tumor-killing efficiency, moving beyond static organoid drug-sensitivity assays by incorporating the critical dimension of immune recruitment and activation.

This personalized methodology holds immediate translational potential, particularly in guiding cancer immunotherapy. Before administering costly and potentially toxic cell therapies, a patient’s tumor organoids can be screened on an OoC platform against different versions of their own engineered CAR-T or TCR-T cells. Readouts such as tumor-killing efficiency, cytokine release, and T cell exhaustion profiles can help identify the most effective and safest therapeutic product for that individual. Moreover, this strategy supports a “clinical trial-on-a-chip” concept. Rather than enrolling large patient cohorts in conventional trials, researchers can create a diverse *in vitro* panel of chips representing various disease subtypes or genetic backgrounds using a biobank of organoids from multiple donors. This approach enables in silico stratification of responders and non-responders, identification of predictive biomarkers, and the study of rare disease subtypes, ultimately de-risking and accelerating the design of future clinical trials.

### 4.2 Coupling with high-resolution multi-omics analyses

While OoCs capture dynamic phenotypes and functional outcomes, the underlying molecular mechanisms often remain unclear. Integrating high-resolution, next-generation sequencing and spatial profiling technologies is essential to uncover these mechanisms, transforming the chip from a physiological model into a powerful discovery tool (103).

#### 4.2.1 *In situ* single-cell RNA sequencing to decode cellular heterogeneity

The cellular material from an OoC experiment is often limited and heterogeneous. Single-cell RNA sequencing (scRNA-seq) is ideally suited to resolve this complexity. After an experiment, for example, a tumor chip treated with an immune checkpoint inhibitor, cells can be harvested from the chip and processed for scRNA-seq. This analysis can reveal distinct transcriptional states across all cell types present: differential gene expression in tumor subpopulations that survived treatment, exhausted versus activated T cell states, and macrophage polarization profiles. By comparing treated and untreated chips, researchers can identify key transcriptional pathways altered by the therapy, providing a mechanistic basis for observed functional outcomes such as tumor killing or resistance.

#### 4.2.2 Spatial transcriptomics and proteomics to resolve cellular interaction networks

A major limitation of bulk or single-cell omics is the loss of spatial context. In immunology, a cell’s physical location, whether adjacent to a tumor cell, embedded in a stromal niche, or circulating in the vasculature, profoundly influences its state. Emerging spatial technologies are now being applied to OoCs to preserve this critical information. Spatial transcriptomics enables genome-wide mapping of gene expression directly on fixed chip sections, revealing, for example, inflammatory signatures in endothelial cells that are in direct contact with adherent immune cells. Multiplexed imaging techniques (*e.g.*, CODEX, MIBI-TOF) can simultaneously label dozens of proteins, visualizing intricate cellular neighborhoods and interaction networks within the engineered tissue. This can reveal spatial correlations between PD-L1^+^ tumor cells and PD-1^+^ T cells, or the proximity of immunosuppressive macrophages to proliferating cancer cells, offering unprecedented insight into the functional architecture of immune responses.

#### 4.2.3 Integrated analysis for novel biomarker and therapeutic target discovery

The utility of these omics approaches is enhanced when datasets are integrated (29, 104, 105). Correlating spatial information from multiplexed imaging with transcriptional profiles from scRNA-seq helps assign specific transcriptional states to cells in defined spatial contexts (106). This multi-modal integration can identify novel, spatially resolved biomarkers of disease progression or treatment response. Furthermore, by analyzing ligand-receptor pairs expressed by interacting cell populations identified through spatial data, researchers can computationally reconstruct intercellular communication networks and predict key signaling pathways driving the observed biology (107). These predictions can then be directly tested and validated by perturbing the same OoC model with specific pathway inhibitors, creating a cycle of hypothesis generation and experimental validation that accelerates the discovery of new therapeutic targets.

In summary, the convergence of IcOCs with patient-specific organoids and high-resolution multi-omics represents a fundamental advance that enhances the utility of these platforms. This integration moves the field from observing immune responses to understanding their molecular and cellular basis, and ultimately to predicting and modulating them for individual patients. This synergistic approach positions IcOCs as an essential tool for both fundamental immunology and the future of precision medicine.

## 5 Current challenges and future perspectives

While significant progress has been made in engineering IcOC platforms, several key challenges must be addressed to fully realize their potential. Moving forward requires not only overcoming technical limitations but also deliberately increasing biological complexity and establishing a clear role for these systems in translational research.

### 5.1 Challenges in standardization and scalability

A major obstacle to the broader adoption of OoC technology, especially in industrial drug discovery, is the lack of standardization. Current platforms often rely on custom designs, proprietary materials, and complex protocols that differ across laboratories, complicating cross-study comparisons and reducing reproducibility. Standardizing platform designs, such as adopting well-plate formats with integrated microfluidics, and simplifying user workflows through automated cell seeding, media exchange, and sampling are essential steps toward creating robust and accessible systems. Additionally, to meet the throughput requirements of pharmaceutical screening, the field must progress beyond single-organ models. Developing medium- to high-throughput screening platforms with arrays of interconnected chips that can be operated and analyzed in parallel is critical for evaluating therapeutic candidates and combinatorial treatments with statistical confidence.

### 5.2 Increasing biological complexity

Advancing organ-on-a-chip technology requires more than technical refinements; it demands a deliberate focus on enhancing biological complexity to achieve physiological fidelity comparable to *in vivo* systems. A key limitation of current single-organ chips is their inability to recapitulate the complete lymphatic return system. This process, essential for maintaining long-term immune memory and peripheral tolerance, involves the drainage of interstitial fluid to lymph nodes. Without it, models cannot accurately emulate antigen capture, presentation, and immune cell recirculation, processes critical for evaluating immunotherapies that target memory cells. Furthermore, the simulation of systemic endocrine regulation, particularly the hypothalamic-pituitary-adrenal (HPA) axis, remains largely absent, limiting the study of neuro-immune-endocrine crosstalk vital for understanding systemic responses in conditions like autoimmune disorders.

Translational discrepancies further highlight these limitations. For example, drug candidates showing promise in chips due to effective local immune modulation may fail in clinical trials because chip models often overlook systemic pharmacokinetics. Similarly, while chips can model local inflammation, they may not fully capture the complex, multi-organ dynamics of severe cytokine storms. This is exemplified by cell-based therapies like CAR-T cells, which can show high efficacy in chips but carry risks like cytokine release syndrome in patients—risks underestimated *in vitro* due to missing systemic feedback loops.

To address this, future platforms must evolve from isolated constructs toward interconnected microphysiological systems. Integrating lymphatic components, microbial communities, and neural elements will be crucial to reconstruct systemic immune regulation, including drainage, neuroendocrine modulation, and microbiome interactions. This shift is necessary to evaluate therapies in a whole-body context, assessing both local efficacy and systemic safety.

Specifically, developing multi-organ systems (“body-on-a-chip”) will enable the study of systemic immune cell trafficking, essential for modeling immunotherapy pharmacokinetics. Incorporating complex microbial communities into organ-specific models will help dissect the microbiome’s role in immune education. Finally, introducing neural and endocrine components will allow the reconstruction of the immune-neuro-endocrine axis to investigate stress-induced immune modulation. Beyond increasing biological complexity, translating insights from these sophisticated models into clinical applications will require rigorous validation and navigation of regulatory pathways.

### 5.3 Validation and regulatory integration

The translational promise of IcOC platforms hinges on rigorous validation against clinical outcomes and their successful integration into regulatory and industrial pipelines. While prospective studies show chip readouts can correlate with patient responses to therapies like chemotherapy, their predictive value for immunotherapies requires further investigation. Enriching models with patient-derived immune components enhances biological relevance but necessitates broader validation to establish inter-laboratory reproducibility for specific disease models.

From a regulatory standpoint, qualifying IcOC assays for use in drug evaluation demands standardized protocols, demonstrated robustness, and a clearly defined “context of use.” This process is increasingly supported by evolving frameworks that recognize alternative, non-animal models. A pivotal consideration for personalized medicine is patient heterogeneity. While patient-derived chips capture individual genetic and immunological profiles, distinguishing general mechanisms from patient-specific variations requires systematic evaluation across diverse cohorts.

Addressing these validation and integration challenges is crucial to solidify the role of IcOCs in translational research and clinical decision-making, ultimately paving the way for their application in personalized immunotherapy.

### 5.4 The path toward translational medicine and personalized immunotherapy

The ultimate validation of IcOC technology depends on its successful integration into drug development and clinical decision-making. This can be realized through three interconnected pathways. First, by generating human-specific efficacy and toxicity data early in the discovery process, IcOCs can serve as a powerful complement to animal testing, bridging the gap between conventional cell assays and *in vivo* studies. This approach helps de-risk drug candidates and may reduce attrition rates in clinical trials.

Second, combining IcOCs with patient-derived organoids and iPSC technology enables the creation of living biobanks of disease models. These patient-specific libraries, representing a range of genetic and phenotypic diversity, will be invaluable for patient stratification, biomarker identification, and predicting individual therapeutic responses, thus anchoring the platform in personalized medicine.

Finally, these human microenvironments offer a unique testbed for accelerating next-generation immunotherapies. They support the evaluation and optimization of novel modalities such as bispecific antibodies and engineered cell therapies, while also enabling assessment of potential adverse events, including cytokine release syndrome, in a controlled, human-relevant setting before clinical use.

In summary, although further development is needed, the trajectory of IcOC technology is well defined. By addressing standardization challenges, incorporating greater biological complexity, and strengthening ties to clinical translation, these biomimetic platforms are positioned to reshape our understanding of human immunology and contribute to the development of safer, more effective immunotherapies.

## 6 Conclusion

In summary, microfluidic engineering serves as a cornerstone for the development of IcOC platforms, representing a significant advance in biomedical research. By enabling precise spatiotemporal control over cellular organization, molecular cues, and biophysical forces, this technology allows researchers to move beyond static observations and actively investigate human immune responses within physiologically relevant tissue environments. These biomimetic systems offer a unique capacity to dissect complex immunological processes, thereby providing new perspectives for drug development. As the field advances through increased standardization and the systematic integration of more complex biological components, these human-based models are positioned to effectively complement or replace certain conventional animal studies. Ultimately, by facilitating the creation of patient-specific models for therapeutic testing, IcOC platforms are expected to accelerate the development of next-generation immunotherapies and contribute to the progression of personalized immuno-medicine.
